# Overexpression of miR-125a-5p Inhibits Hepatocyte Proliferation through the STAT3 Regulation In Vivo and In Vitro

**DOI:** 10.3390/ijms23158661

**Published:** 2022-08-04

**Authors:** Chunyan Zhang, Yabin Zhao, Qiwen Wang, Jianru Qin, Bingyu Ye, Cunshuan Xu, Guoying Yu

**Affiliations:** 1 State Key Laboratory Cell Differentiation and Regulation, Henan Normal University, Xinxiang 453007, China; 2 Henan International Joint Laboratory of Pulmonary Fibrosis, Henan Normal University, Xinxiang 453007, China; 3 Henan Center for Outstanding Overseas Scientists of Pulmonary Fibrosis, Henan Normal University, Xinxiang 453007, China; 4 College of Life Science, Henan Normal University, Xinxiang 453007, China; 5 Institute of Biomedical Science, Henan Normal University, Xinxiang 453007, China; 6 Overseas Expertise Introduction Center for Discipline Innovation of Pulmonary Fibrosis, Henan Normal University, Xinxiang 453007, China

**Keywords:** miR-125a, STAT3, proliferation, liver regeneration, cell cycle

## Abstract

microRNAs (miRNAs) are critically involved in liver regeneration (LR). miR-125a-5p (miR-125a) is a tumor-suppressing miRNA, but its role in LR has not been studied. Our previous studies have proved that miR-125a was related to LR at the initiation phase, while the mechanism hepatocyte proliferation triggered by miR-125a in LR has been rarely evaluated. Herein, we mainly studied the molecular mechanism of miR-125a in triggering hepatocyte proliferation and the proliferation stage of LR. Firstly, a striking reduction of miR-125a was found at 24 h as well as 30 h following partial hepatectomy (PH) in rat liver tissue by miRNAs expression profiles as well as qRT-PCR analysis. Furthermore, in vitro, upregulation of miR-125a decreased proliferation as well as G_1_/S conversion, which promoted hepatocytes apoptosis. STAT3 was the target of miR-125a. In vivo, upregulation of miR-125a by tail vein injection of agomir inhibited LR index. Upregulation of miR-125a inhibited LR index and hepatocytes proliferation by STAT3/p-STAT3/JUN/BCL2 axis. In summary, these current discoveries indicated that miR-125a inhibited hepatocytes proliferation as well as LR by targeting STAT3 and via acting on the STAT3/p-STAT3/JUN/BCL2 axis.

## 1. Introduction

Liver has a remarkable regenerative capacity when it is damaged by injury or surgical resection [1,2,3]. 2/3 PH, also known as 70% PH, refers to the removal of the middle and left lobes of liver that account for 70% of the total liver [4]. After 2/3 PH, hepatocytes instantly enter G_1_ phase, and soon traverse to S phase, this moment the DNA synthesis is up to a peak at 24–48 h after PH [5,6,7]. LR is a synchronized multistep process consisting of initiation (2 to 6 h), proliferation (12 to 72 h) and termination (120 to 168 h) [8,9]. However, the regeneration potential of liver is often impaired by pathological factors, such as liver failure and liver cirrhosis [10,11], thus it is worth emphasizing the necessity of finding novel channels to improve liver’s regenerative potential. 

miRNAs are series of small non-coding RNAs with approximately 20 nucleotides or so, which lead to translational repression or mRNA degradation via negatively regulating their target genes at post-transcriptional level [12,13]. miRNAs take on vital roles in multiple biological fields, in addition to common cell proliferation and apoptosis, as well as metabolism and even carcinogenesis [14,15,16]. Our previous study found some miRNAs were associated with LR at the initiation phase; among them, miR-125a showed a significant up-regulative level at 2 h post-2/3 PH [17]. Recently, several studies have indicated miRNAs take on key roles during the LR [16,18]. For instance, overexpression of miR-21 facilitated liver cell proliferation trough targeting PELI1 [19], FASLG [20] and PTEN [21]; However, down-regulation of miR-378 [22], miR-26a [23] and miR-127 [24] contributed to hepatocytes proliferation during LR. miR-378 was involved in epithelial-mesenchymal transformation (EMT) in Hedgehog-driven regenerative liver cells by targeting GLI-Kruppel family member 3 (Gli3) [22]. In addition, downregulation of miR-23b inhibited cell growth as well as promoting cell apoptosis during the termination phase of LR by activating the TGF-b1/Smad3 pathway [25]; moreover, miR-20a inhibited mouse hepatocyte proliferation and liver regeneration via the TCF4/CDC2/CDC6 axis [26]. These effects of miRNAs on hepatocyte proliferation are relatively basic, and most of them do not involve in vivo mechanism studies. Therefore, it is necessary to excavate underlying mechanisms of other miRNAs in LR. 

In the study, we discovered that miR-125a was markedly decreased in the rat liver tissue at 24 as well as 30 h post-2/3 PH. Subsequently, we illustrated that miR-125a inhibited hepatocytes proliferation, as well as G_1_/S conversion, via the target gene of STAT3, resulting in the inhibition of STAT3/p-STAT3/JUN/BCL2 axis in vivo and in vitro. Conjointly, our study identified miR-125a could act as a suppressor of hepatocytes proliferation as well as LR through regulating of the STAT3/p-STAT3/JUN/BCL2 axis, which might provide a novel target for LR after injury in future.

## 2. Results

### 2.1. miR-125a Was Associated with Rat Liver Regeneration

**Figure 1:**
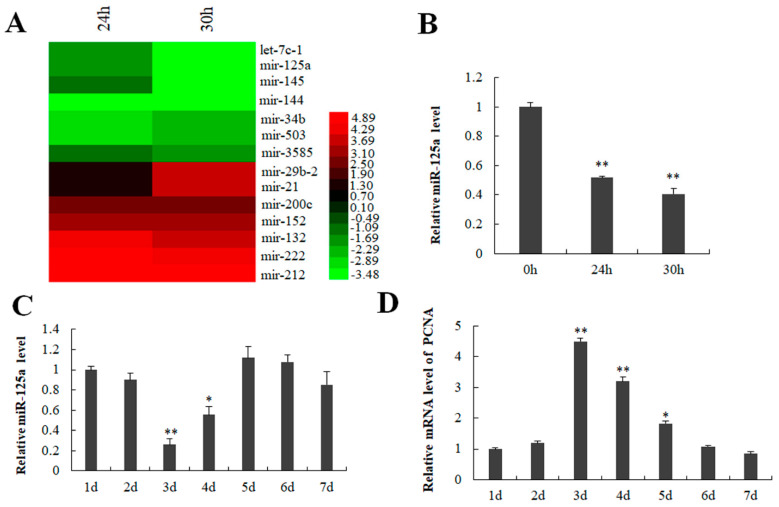
Detection of miR-125a in liver tissue and BRL-3A cells. (**A**) Some abnormally expressed miRNAs are presented by red-green map. (**B**) miR-125a level in liver tissue was verified by qRT-PCR. (**C**) miR-125a level was examined in BRL-3A through qRT-PCR. (**D**). PCNA level was examined through qRT-PCR in BRL-3A cells. Data are shown as mean ±SEM, * *p* < 0.05, ** *p* < 0.01.

It has previously demonstrated that the time 24 h post-PH was a peak of rat hepatocytes proliferation representing via the value of AST as well as BrdU positive cells [27,28]. Here, we established a 2/3 PH model, and analyzed the corresponding biochemical indicators serum aspartate aminotransferase (ALT) and alanine aminotransferase (AST). Compared with the control group. AST and ALT did not increase significantly after PH 24 and PH 30 h (Table S1), which may be related to the recovery of liver function. miRNA expression profiles analysis was used to compare miRNA expression between 24/30 h (post-PH) and 0 h. As a result, miR-29b-2, miR-21, miR-200c, miR-152, miR-132, miR-222 were found to be upregulated, while miR-3585, miR-503, miR-34b, miR-144, miR-145, miR-125a and let-7c-1 downregulated at 24 h and 30 h after PH (Figure 1A). Subsequently, qRT-PCR was utilized to verify the miRNA expression profiles results. As shown in Figure 1B, miR-125a (previous IDs: miR-125a-5p) was sharply decreased at 24/30 h (post-PH). Additionally, miR-125a was also reduced on the 3rd as well as 4th days in hepatocytes post-PH (Figure 1C), which was the hepatocytes logarithmic phase representing via PCNA expression (Figure 1D). Accordingly, research was needed into the function of miR-125a in the proliferation of LR as well as hepatocytes proliferation.

### 2.2. miR-125a Reduced Hepatocytes Proliferation

**Figure 2:**
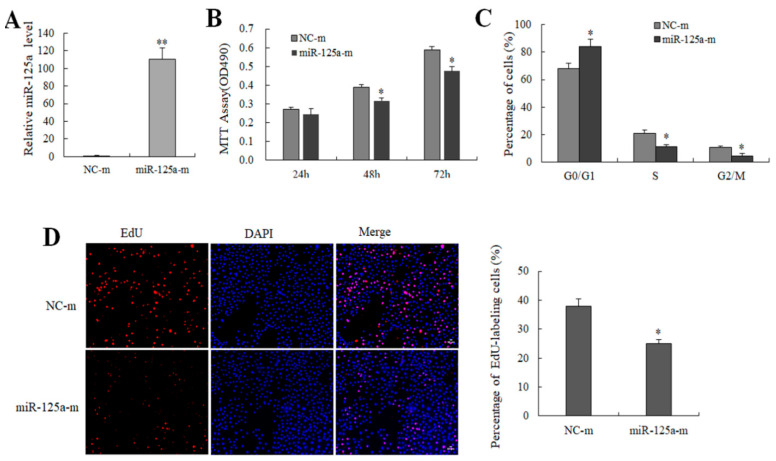
The effect of miR-125a on hepatocytes proliferation (**A**). miR-125a levels were examined through qRT-PCR following transfection of miR-125a mimics (miR-125a-m), and its control NC mimics in BRL-3A cells. (**B**). Cell viability was examined through MTT. (**C**). The role of miR-125a in BRL-3A cell cycles was detected by FACScan. (**D**). Cell proliferation was examined through EdU (red) assay. Data are shown as mean ±SEM, * *p* < 0.05, ** *p* < 0.01.

miR-125a mimics (100 nM) and its control NC mimics were transfected to BRL-3A cells to survey the effect of miR-125a on rat hepatocytes growth as well as proliferation. miR-125a level was increased after the treatment of miR-125a mimics (Figure 2A). Both MTT and EdU assays demonstrated that miR-125a mimics inhibited BRL-3A cells proliferation (Figure 2B,D). However, the opposite results were observed in treatment with an miR-125a inhibitor (Figure S1). Additionally, a cell cycle assay indicated that miR-125a overexpression decreased the transition of G_1_-to-S for BRL-3A cells (Figure 2C and Table S2). Therefore, miR-125a was confirmed to inhibit the BRL-3A cell growth and the proliferation.

### 2.3. miR-125a Induced Hepatocytes Apoptosis

**Figure 3:**
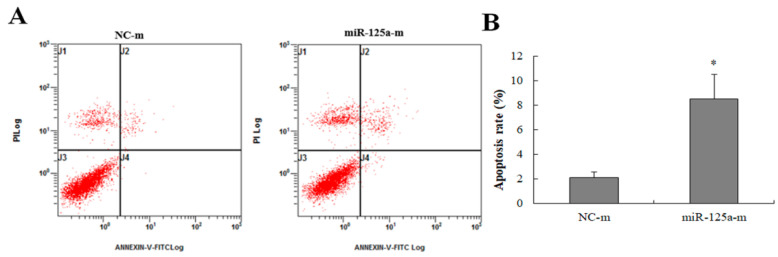
The effect of miR-125a on hepatocytes apoptosis. (**A**).The apoptotic rate was examined through FACScan the after treatment of miR-125a mimics and NC mimics, respectively. J4 (the number of early apoptosis cells) as well as J2 (the number of late apoptosis cells) areas are represented as apoptotic cells. (**B**). Apoptosis rate is represented by a histogram. Data are shown as mean ± SEM, * *p* < 0.05.

To survey the effect of miR-125a on apoptosis, miR-125a mimics and mimics NCs were transfected into BRL-3A cells. As Figure 3A,B demonstrate, the apoptotic rate was increased in mimic groups when compared with NCs group (*p* < 0.05), indicating that miR-125a induced BRL-3A apoptosis. Apoptotic cells were showed in areas of J4 (FITC positive dots showing early apoptosis cells) as well as J2 (V-FITC along with PI double positive dots showing late apoptosis cells) in Figure 3A, suggesting that miR-125a induced hepatocytes apoptosis.

### 2.4. miR-125a Was Negatively Correlated with STAT3 Level In Vivo and Negatively Regulated STAT3 Level In Vitro

**Figure 4:**
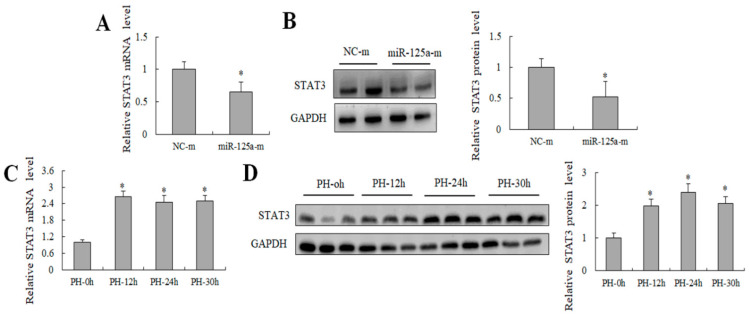
Detection of STAT3 level in vitro and in vivo. (**A**) STAT3 level was examined through qRT-PCR in hepatocytes after treatment of miR-125a reagents. (**B**) STAT3 level were examined through WB in BRL-3A after treatment of miR-125a reagents. (**C**) STAT3 level were examined trough qRT-PCR at 12/24/30 h post-PH (PH-12/24/30 h) in liver tissues. (**D**) miR-125a as well as STAT3 level were examined through WB at 12/24/30 h post-PH (PH-12/24/30 h) in liver tissues. Data are shown as mean ±SEM, * *p* < 0.05.

Previous studies have reported that miR-125a exercises its function in hepatic glycolipid metabolism by targeting STAT3 [29]. Here, the STAT3 expression decreased after treatment with miR-125a mimics in BRL-3A cells (Figure 4A,B). Meanwhile, an opposite trend was found between the STAT3 level and miR-125a level at 12, 24 and 30 h post-PH compared with the control in rat liver (Figure 4C,D), suggesting that there was a possible negative relation between miR-125a and STAT3 during rat LR proliferative stage, STAT3 may be a target gene of miR-125a in rat hepatocytes.

### 2.5. STAT3 Was the Direct Target of miR-125a in Rat Hepatocytes

**Figure 5:**
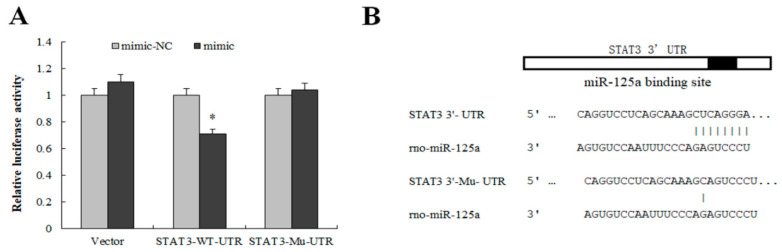
STAT3 was a target gene of miR-125a. (**A**) Luciferase activity was detected by luciferase report assay after co-transfection of STAT3- WT/Mu -UTR as well as miR-125a mimics and NC mimics in BRL-3A. (**B**) The binding site of miR-125a and STAT3- WT/Mu -UTR. Data are shown as mean ± SEM, * *p* < 0.05.

To reveal the effect of miR-125a on rat hepatocytes, GO annotation and KEGG pathway were utilized to categorize the target genes predicted by miRWalk, miRanda, miRDB, miRMap, miRNAMap, RNAhybrid and Targetscan. 2421 putative targets were identified through the above database (Table S3). The JAK/STAT3 signaling pathway was one of the most significantly enriched pathways indirectly related with miR-125a (Table 1), and the target genes were mainly enriched in cell proliferation, death, and apoptosis (Table 2 and Table S4). The activity of the STAT3 3′-UTR luciferase reporter was sharply decreased after treatment of the miR-125a mimic by dual-luciferase assay, but no significant changes were found in mutated STAT3 3′-UTR in the same treatment (Figure 5A,B), suggesting that STAT3 was a direct target of miR-125a in rat hepatocytes. Furthermore, BRL-3A cell proliferation was inhibited after treatment with siRNA of STAT3 (Figure S2).

### 2.6. miR-125a Inhibited Hepatocytes Proliferation through STAT3/P-STAT3/JUN/BCL2 Axis

**Figure 6:**
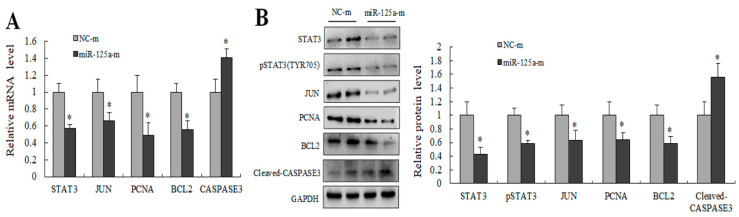
miR-125a inhibited hepatocytes proliferation by STAT3/P-STAT3/JUN/BCL2 axis. (**A**) STAT3, JUN, BCL2, PCNA and CASPASE3 were detected by qRT-PCR in BRL-3A cells after the treatment of miR-125a reagents. (**B**). STAT3, JUN, BCL2, PCNA and Cleaved-CASPASE3 were detected by WB in BRL-3A cells after treatment of miR-125a reagents, the samples derive from the same experiment and that gels/blots were processed in parallel. Data are shown as mean ± SEM, * *p* < 0.05.

Several key genes downstream of STAT3 were examined using qRT-PCR as well as WB for further evaluating the potential mechanism of miR-125a in rat hepatocytes proliferation. Levels of STAT3, p-STAT3, JUN and BCL2 were decreased, and the CASPASE3 level was increased following transfection of miR-125a mimics compared with controls (Figure 6A,B). Collectively, these findings demonstrated that miRNA-125a restrained hepatocytes proliferation by acting on the axis of STAT3/P-STAT3/JUN/BCL2 in vitro.

### 2.7. Expression of miR-125a Agomir in Mouse Liver

**Figure 7:**
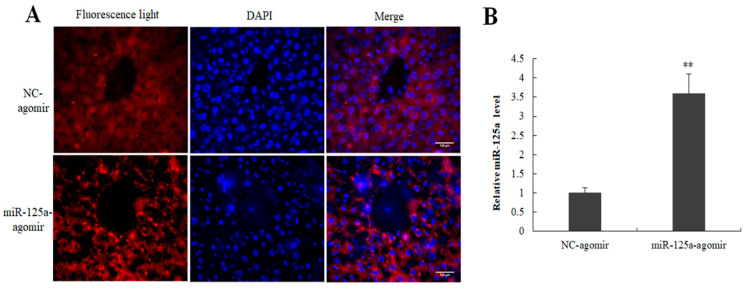
Efficiency detection of miR-125a agomir in mouse liver. (**A**) Cy5-labeled cells were found in miR-125a agomir and its control NC groups by fluorescence microscope. (**B**) miR-125a level was examined through qRT-PCR analysis following injection of miR-125a agomir compared with controls. Data are shown as mean ± SEM. Nuclei were stained with DAPI (blue). Scale bar, 100 μm, ** *p* < 0.01.

To investigate the role of miR-125a in the hepatocytes proliferation of LR, miR-125a agomir and its control NC were injected into mice by tail vein injection 12 h and 24 h before 2/3 PH. 48 h post-2/3 PH, the samples were collected, and qRT-PCR and fluorescence observation were used to examine the expression of miR-125a in mouse liver. Cy5-labeled cells were found in miR-125a agomir and its control NC groups (Figure 7A). The miR-125a level was increased following the injection of miR-125a agomir compared with controls (Figure 7B).

### 2.8. miR-125a Inhibited LR through STAT3/p-STAT3/JUN/BCL2 Axis

**Figure 8:**
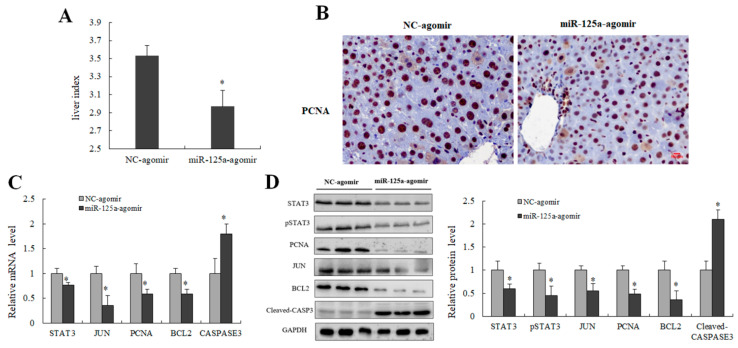
miR-125a inhibited LR through STAT3/p-STAT3/JUN/BCL2 axis in vivo. (**A**) Liver index (liver weight/body weight ratio) was measured at 48 h post-PH after transfection of miR-125a agomir/NC through tail vein injection. (**B**) Representative IHC staining of PCNA (brown) on liver sections obtained from transfection of miR-125a agomir/NC through tail vein injection mice at 48 h post PH; Scale bars: 100 μm. (**C**) STAT3, JUN, BCL2, PCNA as well as CASPASE3 were detected by qRT-PCR in liver tissue after transfection of miR-125a agomir/NC through tail vein injection at 48 h post-PH. (**D**) STAT3, p-STAT3, JUN, BCL2, PCNA as well as Cleaved-CASPASE3 were detected by WB in liver tissue after transfection of miR-125a agomir/NC through tail vein injection at 48 h post-PH, the samples derive from the same experiment and that gels/blots were processed in parallel. Data were shown as mean ± SEM, * *p* < 0.05.

Liver index and PCNA-labeled cells were decreased in injection of the miR-125a agomir group (Figure 8A,B) when compared with controls groups; meanwhile, the levels of key genes downstream of STAT3 including p-STAT3, JUN and BCL2 were decreased, and CASPASE3 level was increased in miR-125a agomir groups (Figure 8C,D). Collectively, these findings demonstrated that miRNA-125a restrained the proliferation process of LR through STAT3/p-STAT3/JUN/BCL2 axis.

## 3. Discussion

2/3 PH offers a unique model to study the mechanism of LR [9]. LR post-PH is primarily aroused via hepatocytes proliferation, which is affected by miRNAs expression [22,25]. At present, the possible mechanisms remain largely unclear. We discovered that some miRNAs, including miR-125a and miR-145, were sharply decreased at 24 and 30 h post-2/3 PH in rat liver tissue through miRNA expression profiles analysis. Herein, we mainly discuss the role of miR-125a in LR and hepatocytes proliferation. The upregulation of miR-125a restrained proliferation as well as G_1_/S transition in hepatocytes. Additionally, miR-125a negatively regulated STAT3 in vitro/vivo. The upregulation of miR-125a restrained mouse liver regeneration in vivo. The following findings illustrated that miR-125a restrained hepatocytes proliferation and LR at the proliferation stage by effecting the axis of STAT3/P-STAT3/JUN/BCL2.

Multiple studies have illustrated that miR-125a played an inhibitory role in several kinds of tumors, such as glioblastoma [30], non-small cell lung carcinoma [31,32,33], hepatocellular cancer [34], retinoblastoma [35], osteosarcoma [36], cervical cancer [37] and breast cancer [38]. In liver, the studies of miR-125a have focused on liver virus infection and hepatocellular carcinoma. It has been reported that miR-125a could interfere with viral replication via binding with surface antigen encoded by its own transcript [39]. Up-regulating miR-125a significantly restrained HCC proliferation as well as metastasis through regulating MMP11 as well as VEGF-A [40]. Additionally, miR-125a-5p along with miR-125b induced the cell cycle blocked at p21-dependent G_1_ phase via suppressing SIRT7 as well as CCND1 level in HCC [41]. Downregulation of miR-125a-5p might protect against isoflurane-induced liver injury by regulating hepatocyte proliferation and apoptosis [42]. miR-125a-5p improved hepatic glucose and lipid metabolism disorders in patients with type 2 diabetes by targeting STAT3 [29]. The above studies suggested that miR-125a may play a significant role in the liver. Here, we discovered miR-125a was sharply downregulated at 24 as well as 30 h post-2/3 PH through miRNA high-throughput sequencing along with qRT-PCR in liver tissues. Previously studies demonstrated that the time of 24 h post-PH was the peak of hepatocytes proliferation, which was shown via a value of serum AST as well as BrdU labeled cells [7]. Moreover, a low level of miR-125a was detected at the logarithmic growth phase of cultivated hepatocytes in the 3rd and 4th days. This indicated that miR-125 may be involved in the proliferation stage of LR. Our subsequent findings also proved that miR-125a restrained cell proliferation, G_1_/S transition, and induced apoptosis in hepatocytes.

It has been proved that the proliferative effect of miRNAs on LR depends on particular target genes of their own [16,17,18,19,20]. Therefore, to further survey the potential regulative mechanism of miR-125a in LR. We screened out cell proliferation-related signaling pathways by GO annotation as well as KEGG pathway assays, such as JAK-STAT3. Interestingly, it was found that STAT3 had an opposite tendency with miR-125a in LR and hepatocytes. Subsequently, the dual-luciferase system along with WB analysis further identified that STAT3 was one of the targets of miR-125a.

STAT3 is a potential transcription factor that can be activated by a series of cytokines and growth factors [43,44]. Activation of STAT3 signal pathway played vital effects on a series of complex life activities including apoptosis, proliferation, invasion, metastasis, differentiation as well as angiogenesis [45,46,47,48]. Additionally, activated STAT3 pathway scould induce abnormal proliferation as well as malignant transformation. Herein, STAT3 has been defined as an oncogene [49]. IL-6 is a pleiotropic cytokine that promotes liver regeneration through the activation of STAT3, and responses to liver injury. IL-6 mediates acute phase responses and induces cytoprotective and mitotic functions [50,51]. In general, IL-6 binds to the interleukin-6 receptor (IL-6R), and the IL-6/IL-6R complex initiates glycoprotein 130 (gp130) for the activation of JAK/STAT, MAPK and PI3K/AKT, which is essential for the early onset of liver disease and the progression and maintenance of the regenerative process [52,53]. STAT3, one of the targets of miR-125a, suppressed tumor invasion as well as metastasis in cervical carcinoma [37]. C-Jun is a major regulator of hepatocyte survival [54] and hepatocyte proliferation during regeneration [55]. It has been illustrated that synergistic activity of STAT3 and c-JUN were observed in human cancer specimens [56,57]. In addition, c-JUN or BCL2 was a downstream target gene of STAT3 in tumorigenesis [58,59,60]. However, whether the STAT3/p-STAT3/JUN/BCL2 axis is administered by miR-125a in hepatocytes and LR at the proliferation stage is unclear. Our findings demonstrated that up-regulation of miR-125a inhibited STAT3, p-STAT3, JUN and BCL2 expression in cultivated hepatocytes and liver tissue.

In conclusion, our current findings showed that miR-125a was presented a low level in the proliferative stage of LR and inhibited the proliferative efficiency of hepatocytes via acting on the STAT3/p-STAT3/JUN/BCL2 axis. Therefore, miR-125a could be served as a potential and novel promising target and regulate the development of LR as well as liver carcinoma.

## 4. Materials and Methods

### 4.1. PH Model Preparation and Tail Vein Injection

Adult Sprague Dawley (SD) rats (male, weighing 230 ± 20 g) were provided by the laboratory animal control office of Henan Normal University. The operation of animal experiments was allowed through Animal Care as well as the Use Committee at the university (License No: HNSD-2020-02-17), and executed sternly in the light of the Animal Protection Law of China. The 2/3 PH models’ operations were executed according to the described method [61]. In short, 30 rats were randomly divided into 5 groups: 2/3 PH groups (PH 24 h) (*n* = 6), 2/3 PH groups (PH 30 h) sham-operated groups (SO 24 h), sham-operated groups (SO 30 h) (*n* = 6), and one control group (0h) (*n* = 6). These were used in miRNA high-throughput sequencing. In addition, another 12 rats were used in miR-125 and STAT3 validation experiments at PH/SO 12 h. Both the 0 h group and sham group were used as controls for miRNA expression profile data analysis as well as miR-125a and STAT3 validation experiments. 

For the vivo studies of miR-125a, 6–8 week Balb/c mice (male, 25 ± 2 g) were purchased from the experimental animal center of Zhengzhou University. The mouse 2/3 PH model was executed according to the method of rat model described above. In short, 12 mice were randomly divided into two groups: the PH-miR-125a agomir group (*n* = 6), and the PH-NC agomir group (*n* = 6). miR-125a agomir (5 nM) and its control agomir NC were injected via tail vein 24 h and 12 h before 2/3 PH operation, respectively. When sampling, the models were sacrificed, and the liver was removed; part of the samples were fixed within 4% paraformaldehyde for immunohistochemistry; part of the samples were embedded with frozen section embedding agent for fluorescence observation; the rest of the samples were stored at −80 °C or liquid azote until further experiment. The agomir was chemically modified and labeled with cy5 for easier cell membrane penetration and fluorescence observation and was purchased from the company (Ribobio, Guangzhou, China).

### 4.2. Fluorescence Observation

The 5-μm-thick slices were obtained from tissue embedded in frozen section embedding agent. After 30 min at room temperature, slices were fixed with acetone and then stained with DAPI for 10 min. Fluorescence microscopy (Axio Imager D2, Carl Zeiss, Germany) was used for photography.

### 4.3. Immunohistochemistry

After deparaffinization/hydration, the 7-μm-thick slices were blocked with endogenous peroxidase blocking solution. They were then treated with 10% normal goat serum (CWBIO, Beijing, China), followed by incubation with the anti-PCNA antibody (1:500, Cell Signaling Technology Cat# 13,110) at 4 °C overnight, and then incubated with Biotin labeled secondary antibody and HRP labeled with streptavidin respectively (CWBIO, China). Finally, slides were stained with 3, 3′-diaminobenzidine and images were captured with a microscope.

### 4.4. Biochemical Index Analysis

After the mice fasted for 12 h, blood was taken from eyelid, 4 °C overnight; blood was centrifuged at 2500 rpm, and then serum was taken and stored at −80 °C. The activity of ALT and AST were executed by ALT/GPT and AST/GOT kits (Wanleibio, Shenyang, China) according to manufacturer’s instruction. Briefly, a 5 μL sample was added into the 20 μL matrix liquid buffer, incubated at 37 °C for 1/2 h. Then, 20 μL of 2, 4-dinitrophenylhydrazine, thoroughly mixed, incubated at 37 °C for 1/3 h was added. Then 200 μL of 0.4 mol/L NaOH, gently shaken, incubated at room temperature for 1/4 h was added. Finally, OD value was determined through a microplate reader at 510 nm.

### 4.5. miRNA High-Throughput Sequencing and Analysis

Total RNA extraction from liver tissue, and miRNA high-throughput sequencing and analysis were conducted as previously described [17,61]. Library preparation and Illumina sequencing were carried out according to Illumina small RNA sample preparation protocol outlined by Shanghai Biotechnology Company (Shanghai, China) [62]. Briefly, firstly, the total liver tissue RNAs were checked and quantified using an Agilent 2100 Bioanalyzer (Agilent Technologies, Santa Clara, CA, USA). Qualified RNA was purified and further linked to the 5′ linker and 3′ linker by T4 RNA ligase, which was then reverse-transcribed into cDNA by the SuperScript II Reverse Transcription Kit (Invitrogen, Carlsbad, CA, USA), which is then amplified and purified. Finally, the purified cDNA library was quantified with a qubit fluorometer (Invitrogen, Carlsbad, CA, USA), and then analyzed by Illumina Genome Analyzer IIx for cluster generation and 36 nt single-end sequencing. The raw data were analyzed as previously described [17]. The difference in miRNA level between PH groups and the control group was considered to be significant at a fold change ≥2 as well as a *p*-value ≤ 0.05.

### 4.6. Cell Culture and Transfection 

Treatments of this part were conducted as our previously described [61]. Briefly, BRL-3A cells were obtained from cell bank of the School of Basic Medicine of Peking Union Medical College (Beijing, China). The cells had been kept in a high glucose DMEM complete medium, supplemented with 10% fetal bovine serum (FBS), 1% penicillin and 1%streptomycin in a concentration of 5% CO_2_ incubator at 37 °C. miR-125a mimics (Ribobio, Guangzhou, China), miR-125a inhibitor (Ribobio, China), or their negative controls handled BRL-3A for 48 h, respectively. To further investigate the role of STAT3 in hepatocyte proliferation, cells were transfected with siRNA-STAT3 (siRNA1, 2, 3, Ribobio, China) or negative control for 48 h, respectively.

### 4.7. MTT Assay

After the cells were transfected with miR-125a mimics and their control, NC mimics, MTT reagent (0.5 mg/mL) was added. After four hours, DMSO was added and dissolved the crystallization; the microplate reader was measured, with OD value at 490 nm. The treatment was operated three times.

### 4.8. EdU Proliferation Assay

After the cells were transfected with miR-125a mimics and its control NC mimics, respectively, EdU (50 μmol/L, Ribobio, Guangzhou, China) was added and incubated for 2 h at 37 °C. Subsequently, 4% paraformaldehyde was used for fixing for 1/2 h at 4 °C. Subsequently Triton X-100 (0.5%) was used for permeabilization for 10 min. Finally, after 1 × Apollo^®^ reaction cocktail treating for 1/2 h, DAPI was utilized for staining for 1/2 h. Fluorescence microscopy was used for photography.

### 4.9. Cell Cycle Analysis

After the cells were transfected with miR-125a mimics and its control NC mimics for 48 h, respectively, the transfected BRL-3A cells were harvested. After fixing overnight at least, PI (20 μg, Sigma, Saint Louis, MI, USA) and RNase A (50 μg, Sigma, Saint Louis, MI, USA) in 1 mL PBS solution were used for treating without light for 0.5 h at 37 °C. Then DNA content was measured by FACSCan.

### 4.10. Cell Apoptosis

After the cells were transfected with miR-125a mimics and its control NC mimics for 48 h, respectively, the transfected BRL-3A cells were harvested. 1× Binding Buffer was used for washing and resuspending cells. Next, Annexin-V-FITC (3.5 μL, BD, Franklin Lakes, NJ, USA) and PI (3.5 μL, BD, USA) in 100 μL Binding Buffer was added and cells were incubated without light for 1/2 h. Finally, 1× Binding Buffer (400 μL) was added to stop the reaction, followed by FACSCan as quickly as possible.

### 4.11. Luciferase Vector Acquisition and Detection

Firstly, 3′UTR of STAT3 containing the recognition site by miR-125a was amplified and inserted into psiCHECK-2 (Promega, Madison, WI, USA). Meanwhile, the corresponding mutant 3′UTR of STAT3 without a corresponding recognition site was amplified and inserted into the same empty vector. Next, BRL-3A cells were plated overnight, then co-treated with miR-125a reagents as well as the recombinant WT/Mut-vector. Finally, a dual-luciferase kit (Promega, Madison, WI, USA) was used to obtain luciferase activities.

### 4.12. RNA Acquisition and qRT-PCR

RNA acquisition as well as cDNA synthesis was performed in RNA isolation reagent (Invitrogen, Carlsbad, CA, USA) and cDNA synthesis kit (Promega, Madison, WI, USA), respectively. The detail qRT-PCR instruction was described in our previous study [61]. Information of the primers used is summarized in Table 3. U6 or GAPDH was used as the internal reference to normalize miRNA and total mRNA level. Finally, the relative quantitative method of 2^−ΔΔCt^ was applied to calculate the levels of genes under test.

### 4.13. Western Blot Analysis

After extracting proteins, as well as measuring the concentration, SDS-PAGE was used to separate protein. Then, the separated proteins were transferred to the NC membrane (GE, Newark, DE, USA). Subsequently, different rabbit antibodies (1:1000), including anti-STAT3 (Boster, Wuhan, China), anti-p-STAT3 (Boster, Wuhan, China), anti-JUN (Bioss, Beijing, China), anti-BCL2 (Boster, Wuhan, China), anti-PCNA (CST, MA, USA) and anti-CASEPASE3 (Boster, Wuhan, China), were used to incubate the membrane for 16 h (4 °C). Next, a secondary IgG antibody against rabbits (1:5000) (Sigma, MI, USA) was used to incubate the membrane for 1 h (37 °C). Finally, ECL reagents were used for visualization of the band. The Image Quant system (ImageQuant LAS 4000, GE, Newark, DE, USA ) was used for measuring the band. GAPDH (1:3000) (Sigma, MI, USA) acted as an internal control. 

### 4.14. Data Analysis

SPSS software version 18.0 (SPSS Inc, Chicago, IL, USA) was applied to deal with data. Mean ± SEM were used to show data. The difference between groups was analyzed by an independent t-test or an ANOVA-containing post hoc test. The value of *p* < 0.05 showed statistical significance.

## Figures and Tables

**Table 1:** Pathway enrichment analysis of the predicted target genes of miR-125a.

Enriched KEGG Pathways	*p*-Value	Genes
JAK-STAT signaling pathway	0.0066	CSF3, PTPN6, GRB2, IL6ST, CTF1, IL6R, IL24, BCL2L1, STAT3, IL11, LEP, PIAS3, IL4R, SOS2, PIK3CA, PIK3R5, IFNGR2, EPO, THPO
Proteoglycans in cancer	0.0089	CDX2, THRB, MED22, RFXANK, MED20, CBFB, TAF7L, FOXS1, POU4F3, CTDSP1, RBPJL, ZFP518A, TBL1XR1, FOXJ1, LDB1, TADA2B, MLXIPL, FOXN1, GRHL3, GRHL2, NRIP1, BRWD1, HNF4A, MED15, ETV3L, VEGFA, ZFP395, TADA3, TFCP2L1, ABCA2, ZFP110, MSX2, TAL2, FOXH1, VDR, NPAS1, MSX3, FOXQ1, ELK4, OVOL1, TFDP2, ETV4, SIM1, EPO, RFX5, ZMYM3, FOXA1, KLF16, MAFK, FOXP3, ZFP444, SNAI1, STAT3, SOD2, NOTCH1, SP1, ETS1, MAPK14, LOC100911917, MLX, BNC1, KDM4C
Insulin resistance	0.0216	PPARA, MLXIPL, CREB5, PPP1R3A, CPT1A, STAT3, PPP1R3D, TNFRSF1A, PPP1CA, RPS6KA1, RPS6KA2, MLX, GYS1, PIK3CA, SLC27A6, PIK3R5, PTPN1, SLC27A4
HIF-1 signaling pathway	0.0263	FLT1, EDN1, MKNK2, MKNK1, IL6R, STAT3, EIF4EBP1, TFRC, PLCG1, VEGFA, SERPINE1, PIK3CA, PIK3R5, CAMK2B, EGF, IFNGR2, EPO

**Table 2:** Functional enrichment analysis of the predicted target genes of miR-125a.

Enriched Biological Processes	No. of Genes	*p*-Value
negative regulation of apoptotic process	70	0.00323
positive regulation of transcription, DNA-templated	71	0.02048
negative regulation of neuron death	11	0.03945
negative regulation of cell death	15	0.04121
positive regulation of cell proliferation	61	0.04763
positive regulation of cell migration	37	0.00028
positive regulation of apoptotic process	45	0.02398
negative regulation of intrinsic apoptotic signaling pathway	8	0.01467
positive regulation of JUN kinase activity	10	0.01670
angiogenesis	30	0.00546
positive regulation of apoptotic signaling pathway	10	0.01030
cell migration	30	0.01378
positive regulation of MAP kinase activity	12	0.01442

**Table 3:** Primers used in reverse transcription and quantitative real-time PCR.

miRNA and Genes	Pimers Sequences (5′→3′)
miR-125a RT	GTCGTATCCAGTGCAGGGTCCGAGGTA TTCGCACTGGATACGACTCACAG
miR-125a FP	TCCCTGAGACCCTTTAACCT
miR-125a RP	GTGCAGGGTCCGAGGT
U6 FP	CTCGCTTCGGCAGCACA
U6 RP	AACGCTTCACGAATTTGCGT
r-STAT3 FP	GTGGAAAAGGACATCAGTGGCA
r-STAT3 RP	CTTGGTCTTCAGGTAAGGGGCA
r-JUN FP	GGCTGTTCATCTGTTTGTCTTCAT
r-JUN RP	CCCTTTTCTTTACGGTCTCGGT
r-CASPASE3 FP	GAGCTGGACTGCGGTATTGAG
r-CASPASE3 RP	AACCATGACCCGTCCCTTGA
r-BCL2 FP	CGACCTCTGTTTGATTTCTCCTG
r-BCL2 RP	CTTTTCATATTTGTTTGGGGCA
r-PCNA FP	GGGTGAAGTTTTCTGCGAGTG
r-PCNA RP	GGAGACAGTGGAGTGGCTTTT
r-GAPDH FP	AAGATGGTGAAGGTCGGTGTGA
r-GAPDH RP	TCGCTCCTGGAAGATGGTGAT
m-STAT3 FP	AACCTCCAGGACGACTTTGATTT
m-STAT3 RP	GTTTCTTAATTTGTTGGCGGGTC
m-JUN FP	CAGAGTTGCACTGAGTGTGGC
m-JUN RP	GCAGTTGGTGAGAAAATGAAGAC
m-CASPASE3 FP	GTCTGACTGGAAAGCCGAAACTCT
m-CASPASE3 RP	AAAGGGACTGGATGAACCACGAC
m-BCL2 FP	GCCACCTGTGGTCCATCTGA
m-BCL2 RP	GAGACAGCCAGGAGAAATCAAAC
m-PCNA FP	TTGCACGTATATGCCGAGACC
m-PCNA RP	GGTGAACAGGCTCATTCATCTCT
m-GAPDH FP	TGGCCTTCCGTGTTCCTAC
m-GAPDH RP	GAGTTGCTGTTGAAGTCGCA

## Data Availability

All data supporting the findings of this study appear in the submitted manuscript or are available from the corresponding author upon reasonable request.

## Supplementary Materials

The following supporting information can be downloaded at: https://www.mdpi.com/article/10.3390/ijms23158661/s1.
