# Supplementary material for: Overexpression of miR-125a-5p Inhibits Hepatocyte Proliferation through the STAT3 Regulation In Vivo and In Vitro
Source: Int J Mol Sci. 2022 Aug 4;23(15):8661. doi: 10.3390/ijms23158661 (PMC9369155; doi:10.3390/ijms23158661)

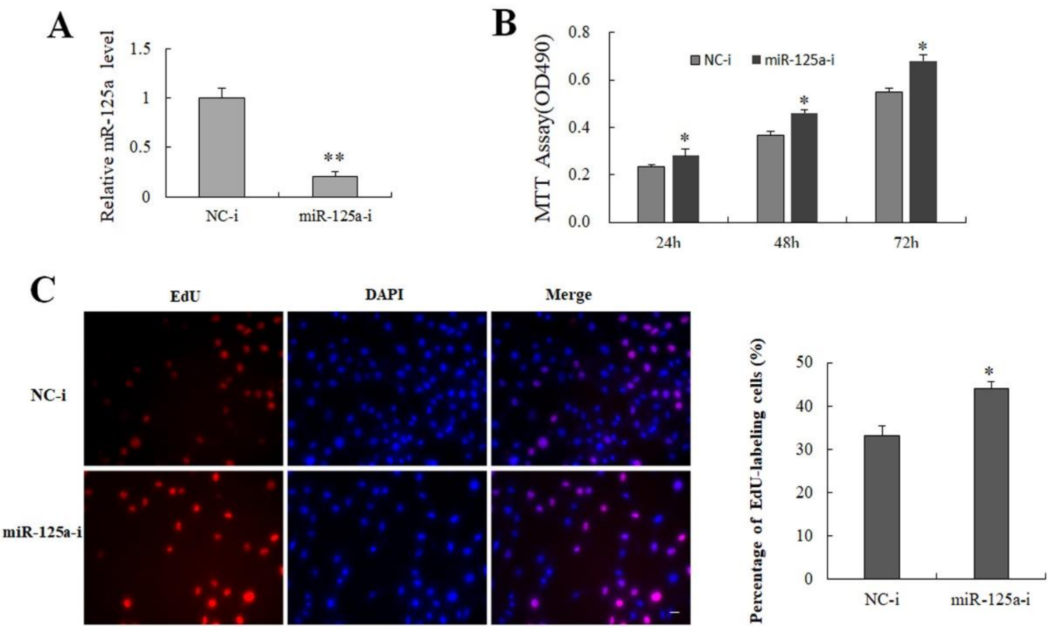

Supplementary figure S1. The effect of miR-125a inhibitor on hepatocytes proliferation. A. miR-125a level were examined through qRT-PCRs analysis following transfection of miR-125a inhibitors (miR-125a-i), and its control NC inhibitors (NC-i) in BRL-3A cells. B. Cell viability were examined through MTT. C. Cell proliferation were examined through EdU (red) assay. Scale bar, 50 µm. Data were shown as mean ±SEM, *p < 0.05, **p < 0.01.

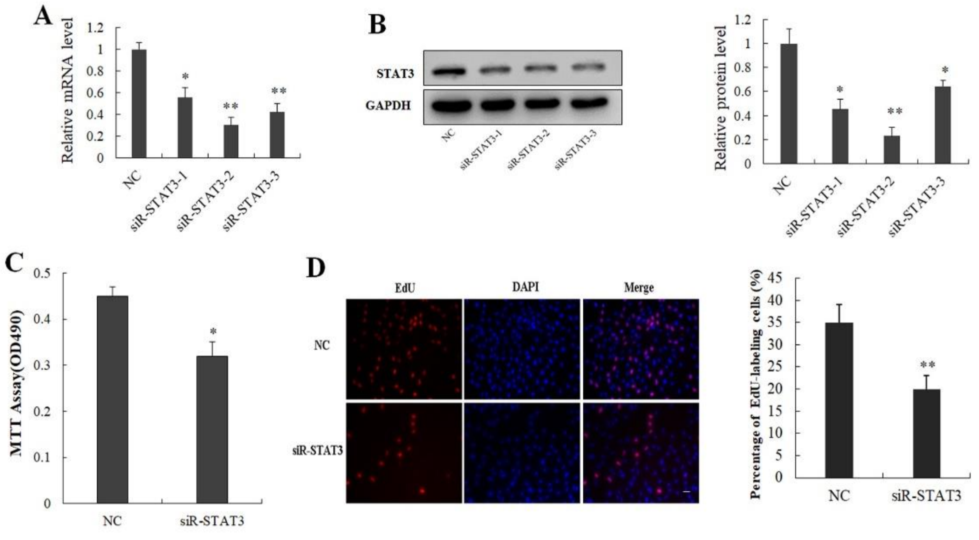

Supplementary figure S2. The effect of knockdown STAT3 on hepatocytes proliferation A. STAT3 level were examined through qRT-PCRs analysis following transfection of siRNAs of STAT3(siRNA1, 2, 3), and its negative control (NC) in BRL-3A cells. B. A. STAT3 level were examined through WB analysis following transfection of siRNAs of STAT3, and its negative control (NC) in BRL-3A cells. C. Cell viability were examined through MTT following transfection of siRNA2 of STAT3. D. Cell proliferation were examined through EdU (red) assay following transfection of siRNA2 of STAT3. Scale bar, 50 µm. Data were shown as mean ±SEM, *p < 0.05, **p < 0.01.

The original gel image of supplementary figure S2B

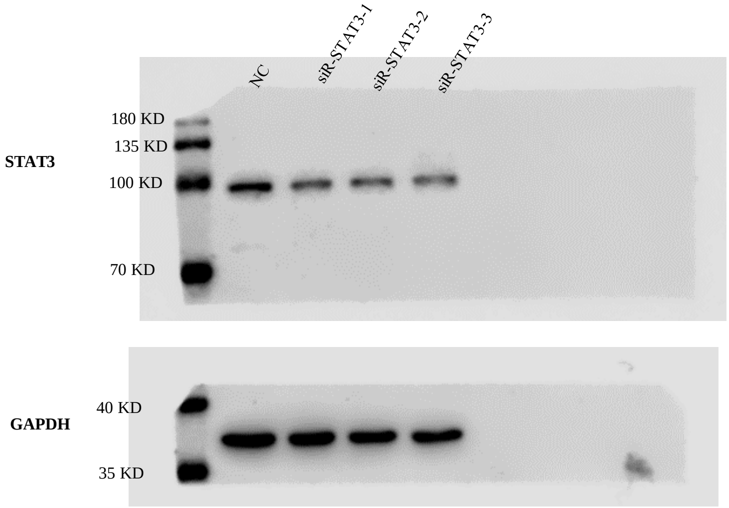

Supplement: Supplementary file 1 [file ijms-23-08661-s001.zip › Supplementary figuresú¿including origin gel image of Supplementary fig2B ú⌐.pdf]
