# Supplementary material for: Overexpression of miR-125a-5p Inhibits Hepatocyte Proliferation through the STAT3 Regulation In Vivo and In Vitro
Source: Int J Mol Sci. 2022 Aug 4;23(15):8661. doi: 10.3390/ijms23158661 (PMC9369155; doi:10.3390/ijms23158661)
Supplement: Supplementary file 1 [file ijms-23-08661-s001.zip › supplementary Table S4.pdf]

Supplementary Table S4. Functional enrichment analysis of the predicted target genes of miR-125a

**Enriched biological processes**	**genes**	***P*-value**
Negative regulation of apoptotic process	PPARD, MAEA, HMGCR, FIGNL1, MMP9, ARNT2, NAA15, TP63, AURKA, AQP1, TRIAP1, BDNF, ATAD3A, CASP3, MYD88, ATG5, SLC25A27, FAM129B, NQO1, SOX10, NOL3, IL24, PIM2, THY1, PRAMEF12, CD38, KDM2A, BTG2, VEGFA, PDGFRB, WNT11, SIAH2, NGFR, MYO18A, CAMK1D, ARL6IP1, LOC684466, TAF9B, PRKDC, HSPA1A, BCL2L2, BCL2L1, HSPA1B, SRC, MSX2, TNFRSF1A, CHD8, STK40, DDX3X, PTK2B, LHX3, HSPA5, EPO, LTK, HCK, TGFBR1, GAS6, STAT3, SOD2, KDR, LEP, PPIF, PLK3, RPS6KA1, AREL1, CD59, ABO, WNT7A, GRK1, CBS	0.00323
Positive regulation of transcription, DNA-templated	PPARA, PPARD, GDF2, CDX2, WNT3A, ARNT2, NAA15, TP63, FOXO4, BRPF1, SERTAD3, ATOH8, PLCB1, RBPJL, CIITA, SOX10, TBL1XR1, RET, PCBD1, MLXIPL, BANP, GRHL3, GRHL2, FOXN3, CD38, MTF1, HNF4A, HIPK2, MYRF, WNT11, HAS3, MAPRE3, ING5, TADA3, CRLF3, SRF, SRC, CXXC1, FOXH1, CHD8, LHX3, HINFP, EGF, FBXW11, ETV4, EPO, DVL2, ESRRA, MAP2K3, TGFBR1, HMBOX1, ESRRG, CREB5, FOXP3, USF1, SNAI1, ATMIN, STAT3, DVL1, SREBF2, NOTCH1, RPS6KA1, SP1, ETS1, KDM8, ZFP281, PHF5A, IRF4, KLF1, WNT7A, TP53INP2	0.02048
Negative regulation of neuron death	CSF3, PPARA, BDNF, NOS1AP, SORL1, VEGFA, PSMC1, NEO1, STAT3, EPO, PPP5C	0.03945
Negative regulation of cell death	HSPA1A, TEAD2, HSPA1B, STAT3, NOTCH1, TMEM109, BDNF, ATG5, SRSF6, CTGF, MCFD2, VPS4B, WNT11, WNT9A, PPP5C	0.04121
Positive regulation of cell proliferation	CDX2, THRB, HMGCR, IL6ST, WNT3A, ARNT2, EDN1, CXCL12, IL11, AKR1C2, CTGF, SLC25A27, UBE2A, CHP2, MLXIPL, IL6R, IL24, SPDYA, EAPP, PRAMEF12, TNS3, HIPK1, RRM2, HIPK2, VEGFA, PDGFRB, LAMC2,	0.04763

	CARM1, EIF5A2, RGD1564379, EMP2, CSF3, FGFR2, KMT2D, GCNT2, BCL2L1, PTK2B, PRKRA, RTKN2, EGF, THPO, EPO, COL18A1, PTPN6, ACER3, FLT1, HCK, ACER2, TGFBR1, ZFP703, STAT3, KDR, LEP, NOTCH1, S100B, ETS1, NTRK2, KDM4C, ABO, WNT7A	
Positive regulation of cell migration	CCL3, GCNT2, EDN1, CBLL1, AQP1, CXCL12, SEMA5A, PTK2B, SEMA3F, SEMA3E, ADRA2A, SEMA3C, HSPA5, FAM83H, COL18A1, RET, FLT1, ARHGEF39, PDPN, PODXL, TGFBR1, ZFP703, SUN2, SNAI1, KDR, SPAG9, NOTCH1, SEMA4G, ETS1, SEMA4F, VEGFA, SEMA4C, PDGFRB, SEMA4B, LAMC2, WNT11, SEMA4D	0.00028
Positive regulation of apoptotic process	CDK19, ING5, BCLAF1, MMP9, ZMAT3, PRKDC, BCL2L1, LATS1, SRC, MSX2, BAK1, BDNF, CASP3, DDX3X, MTCH2, MAP3K1, PPP2CA, UNC5C, INPP5D, CASP2, ANO6, BMF, LTA, EIF2B5, ZFP346, TXNIP, DAB2IP, ARHGEF7, DFFA, IL24, ATF6, LOC100911911, TRIM35, NOTCH1, RASSF6, S100B, RPS6KA2, BBC3, MAP3K10, PDGFRB, WNT11, NGFR, MAP3K11, CAMK1D, DUSP6	0.02398
Negative regulation of intrinsic apoptotic signaling pathway	PPIF, NOL3, DDX3X, FIGNL1, MMP9, BCL2L2, BCL2L1, SRC	0.01467
Positive regulation of JUN kinase activity	DVL2, TRAF2, EPHA4, DAB2IP, PTK2B, MAP3K1, EDN1, MAP3K10, TRAF6, MAP3K11	0.01670
Angiogenesis	FGFR2, CAV1, GDF2, ANPEP, ENPEP, EPHB4, GJA5, WARS, TSPAN12, CTGF, PTK2B, SERPINE1, PIK3CA, ADRA2B, EGF, VEZF1, RAMP1, SYK, COL18A1, DAB2IP, FLT1, TGFBR1, TBX4, THY1, KDR, LEP, DLL4, MAPK14, VEGFA, WNT7A	0.00546
Positive regulation of apoptotic signaling pathway	ING5, DAB2IP, ADORA2A, TGFBR1, TP63, CAMK2B, NGFR, BMF, CASP2, TRIM39	0.01030
Cell migration	APC2, SORL1, ASTN1, PTK7, RASGEF1A, ARHGAP35, ENPEP, CXCL12, SRC, CTGF, PAFAH1B1, ADAM9, ARC, FLT1, PODXL,	0.01378

	FSCN1, VAV2, SNAI1, KDR, JUP, PPP1R9B, TNS3, SDC1, PLCG1, ITGA7, VEGFA, GFRA1, PDGFRB, EMP2, MYO18A	
Positive regulation of MAP kinase activity	AJUBA, FLT1, DIRAS2, MAP3K1, EDN1, VEGFA, ADRA2A, PDGFRB, PIK3R5, EGF, CEACAM1, SRC	0.01442
